# Early Warning Signals for Regime Transition in the Stable Boundary Layer: A Model Study

**DOI:** 10.1007/s10546-016-0199-9

**Published:** 2016-10-11

**Authors:** I. G. S. van Hooijdonk, A. F. Moene, M. Scheffer, H. J. H. Clercx, B. J. H. van de Wiel

**Affiliations:** 1 Fluid Dynamics Laboratory and J.M. Burgerscentrum, Eindhoven University of Technology, De Rondom 70, 5612 AP Eindhoven, The Netherlands; 2 Department of Meteorology and Air Quality, Wageningen University and Research Centre, Droevendaalsesteeg 4, 6708 PB Wageningen, The Netherlands; 3 Department of Aquatic Ecology and Water Quality Management, Wageningen University and Research Centre, Droevendaalsesteeg 4, 6708 PB Wageningen, The Netherlands; 4 Department of Remote Sensing and Geosciences, Delft University of Technology, Stevinweg 1, 2628 CN Delft, The Netherlands

**Keywords:** Early warning signals, Critical regime transition, Maximum sustainable heat flux, Nocturnal boundary layer, Minimum wind speed

## Abstract

The evening transition is investigated in an idealized model for the nocturnal boundary layer. From earlier studies it is known that the nocturnal boundary layer may manifest itself in two distinct regimes, depending on the ambient synoptic conditions: strong-wind or overcast conditions typically lead to weakly stable, turbulent nights; clear-sky and weak-wind conditions, on the other hand, lead to very stable, weakly turbulent conditions. Previously, the dynamical behaviour near the transition between these regimes was investigated in an idealized setting, relying on Monin–Obukhov (MO) similarity to describe turbulent transport. Here, we investigate a similar set-up, using direct numerical simulation; in contrast to MO-based models, this type of simulation does not need to rely on turbulence closure assumptions. We show that previous predictions are verified, but now independent of turbulence parametrizations. Also, it appears that a regime shift to the very stable state is signaled in advance by specific changes in the dynamics of the turbulent boundary layer. Here, we show how these changes may be used to infer a quantitative estimate of the transition point from the weakly stable boundary layer to the very stable boundary layer. In addition, it is shown that the idealized, nocturnal boundary-layer system shares important similarities with generic non-linear dynamical systems that exhibit critical transitions. Therefore, the presence of other, generic early warning signals is tested as well. Indeed, indications are found that such signals are present in stably stratified turbulent flows.

## Introduction

The collapse of turbulence is investigated here using a direct numerical simulation (DNS) of a Couette flow with constant heat-flux boundary conditions. This idealized set-up is used as a model for the nocturnal boundary layer (NBL). Of special interest are changes in the dynamical behaviour as the system approaches a regime transition, that is the collapse of turbulence. We show that certain properties of the flow signal the imminence of a critical regime transition, and we demonstrate that those ‘early warnings’ may be used to estimate the transition point beforehand. An important aspect of the method is that it does not rely on a parametrized description of turbulence.

In many studies a qualitative distinction is made between the weakly stable boundary layer and the very stable boundary layer (e.g. Mahrt 1998; Fernando and Weil 2010; Monahan et al. 2011; Sun et al. 2012; Mahrt 2014; Bonin et al. 2015; Acevedo et al. 2015; Hooijdonk et al. 2015). The weakly stable boundary layer (WSBL) generally occurs when winds are strong or when clouds are present. In this case, turbulence is relatively intense, strong and continuous (e.g. Sun et al. 2004; Sorbjan 2015). As such, classical theories for turbulence, that is Monin–Obukhov (MO) similarity theory (Monin and Obukhov 1954) or local scaling (Nieuwstadt 1984), generally seem to be applicable in relating turbulence quantities to mean flow properties (e.g. Grachev et al. 2005; Sorbjan 2006; Beare et al. 2006; Fernando and Weil 2010; Svensson et al. 2011; Mahrt 2014).

In contrast, the very stable boundary layer (VSBL) generally occurs after sunset under a combination of weak winds and a clear sky. Under such conditions, significant surface cooling occurs, while weak winds are incapable of mixing against buoyancy forces (e.g. Derbyshire 1999; Wiel et al. 2012a, b). As a result of intensified stratification, turbulence may collapse to a very weak state (Businger et al. 1971; Sun et al. 2012; Wiel et al. 2012a, b; Mahrt 2014; Mahrt et al. 2015), and flow in the upper part of the boundary layer may become decoupled from the surface (e.g. Derbyshire 1999; Mahrt 1999; Acevedo and Fitzjarrald 2003; Williams et al. 2013; Donda et al. 2015a). At the same time, it is unlikely that complete laminarization will occur in atmospheric flows even at large stability (Mauritsen et al. 2007; Zilitinkevich et al. 2008). In such ‘collapsed’ cases, the main contribution to turbulent transport often arises from intermittent bursts (Nappo 1991; Wiel et al. 2003; Ansorge and Mellado 2014; Sun et al. 2015; He and Basu 2015). In turn, these bursts may (temporarily) recouple the boundary-layer flow to the surface (e.g. Wiel et al. 2003; Sun et al. 2004. Terrain slope and inhomogeneity may also significantly affect the flow in this regime (e.g. Shapiro and Fedorovich 2007; Stoll and Porté-Agel 2009; Viana et al. 2012; Mahrt et al. 2013). As a result, modelling the VSBL remains a challenge since classical scaling laws may be inapplicable (e.g. Fernando and Weil 2010; Holtslag et al. 2013; Mahrt 2014).

The division into the WSBL and VSBL itself can be physically understood from the so-called maximum sustainable heat flux (MSHF) theory (Wiel et al. 2007, 2012a, b). An important insight is that the downward turbulent heat flux is limited to a maximum value. Qualitatively, this must exist because the turbulent heat flux becomes small in both the neutrally stratified limit (small gradients) and in the strongly stratified limit (weak turbulent mixing). A quantitative expression for the MSHF can be obtained within the MO framework, and it can be shown that the value of the maximum is related to the cube of the ambient wind shear (e.g. Malhi 1995). Observational evidence confirms the existence of such a maximum (e.g. Mahrt 1998; Basu et al. 2006; Sorbjan 2006; Monahan et al. 2015; Hooijdonk et al. 2015).

The division into two regimes is based on whether or not the surface energy budget is in balance (Wiel et al. 2012a; Hooijdonk et al. 2015). Consider, for example, a low-heat-capacity surface (e.g. covered with fresh snow) with a fixed surface energy loss (i.e. due to the emission of longwave radiation). To reach a balance in the surface energy budget, turbulent heat transport must supply the same amount as the radiative energy loss towards the surface (or at least a significant fraction thereof). Based on the wind speed, we can now distinguish between two regimes: if winds are strong, that is the MSHF is large, the boundary layer quickly adjusts, such that the downward heat transport balances the surface energy loss. This case is classified as being ‘weakly stable’. On the other hand, if winds are weak, no such balance can be reached since even the MSHF is insufficient to compensate for surface energy loss. Consequently, temperature gradients increase, which in turn further limits the downward heat flux. Generally, this positive feedback leads to strongly stratified (‘very stable’) conditions (e.g. such as observed in Mahrt 2011).

Following from these two limit cases, a threshold wind speed can be formulated, that is the minimum wind speed for which the surface energy budget can be balanced by downward heat transport (Wiel et al. 2012b). Below the threshold, turbulence is very weak, while above the threshold, turbulence is relatively strong and continuous. Several observational studies indeed show a threshold wind speed for the existence of continuous turbulence (e.g. King 1994; Sun et al. 2012; Acevedo et al. 2015). More recent studies provide compelling observational evidence for the division into two regimes based on MSHF theory (Hooijdonk et al. 2015; Monahan et al. 2015; Bonin et al. 2015). Monahan et al. (2015) applied advanced statistical analysis to field observations, a so-called hidden Markov model, to demonstrate the existence of two distinct regimes. Additionally, idealized numerical studies also show that a flow transition occurs for supercritical surface cooling (Nieuwstadt 2005; Flores and Riley 2011; Donda et al. 2015b). As such, it appears the MSHF framework indeed explains the physical mechanism that warrants the division into two regimes.

The existence of a maximum heat flux was shown in dynamical single-column models based on idealized physical arguments and the application of MO similarity (e.g. Derbyshire 1999; Wiel et al. 2012a). Other studies investigated the dynamic stability of equilibria of such models (McNider et al. 1995; Wiel et al. 2007). Although such one-dimensional models provide important insights using turbulence parametrizations, it remains unclear how these aspects of multiple equilibria and dynamic stability manifest themselves in a fully resolved three-dimensional flow. A novel aspect of our study is the application of direct numerical simulation (DNS) to replicate the idealized NBL model as set up by Wiel et al. (2007) in a three-dimensional setting. As in DNS, the Navier–Stokes equations are solved up to the Kolmogorov length scale; we do not rely on the validity of any parametrization for turbulence, for example MO theory, at any stage. We specifically study aspects of the system related to the dynamically stable equilibrium. Increased understanding on a fundamental level may be beneficial for the interpretation of field observations, and additionally, a well-understood idealized system can serve as a canonical case.

Although MSHF theory explains the existence of two regimes, it remains a challenge to predict the transition point. In parametrized models a transition from a turbulent flow (WSBL regime) to a laminar flow (VSBL regime) can be predicted using MO similarity. Without such parametrizations, however, such predictive metrics do not exist. We investigate the predictability of a regime transition in Couette flow when surface cooling is systematically increased. Two novel approaches are employed to obtain such early warning signals for regime transition, without relying on turbulence parametrization. First, MSHF theory suggests that changes in flow characteristics (e.g. the temperature signal) may indicate an imminent regime transition. We investigate if these system-specific early warning signals are present in our set-up. Second, we use dynamical systems theory to investigate the presence of generic signals, which are applied in other fields (e.g. Scheffer et al. 2001; Veraart et al. 2012; Tantet 2016). These approaches may provide potential tools for studying critical regime transitions in turbulent flows.

The paper is organized as follows. In Sect. 2, the current model is described and in detail, and in Sect. 3 the numerical method and research strategy are discussed and validated. Section 4 is divided into two main parts; first, results are presented that show how the system responds to different surface cooling rates. Next, we show how the results can be used to infer a closure-independent estimate of the critical point. Section 5 contains the discussion and conclusions, and the paper is finalized with a brief summary and outlook.

**Fig. 1:**
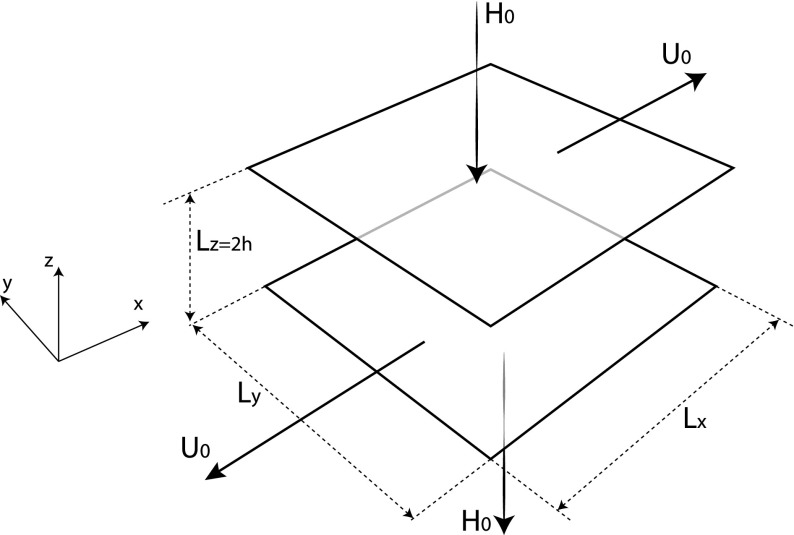
Sketch of model set-up. The two parallel plates (no-slip boundary conditions) at distance $L_z=2h$ move in opposite directions at velocity $U_0$. A negative (i.e. downward) heat flux (Neumann boundary conditions) is applied at the bottom and top boundaries. In horizontal directions the domain is periodic

## Model set-up

### Couette flow

The Couette flow is driven by two horizontally oriented parallel plates, at a vertical distance 2*h* (Fig. 1), where the parallel plates move in opposite direction with velocity $\pm U_0$. For this set-up the streamwise, spanwise and vertical directions are defined as *x*,  *y* and *z*, respectively. We define the Reynolds number for this flow as

1 $$\begin{aligned} Re = \frac{U_0 h}{\nu }, \end{aligned}$$

where $\nu$ is the kinematic viscosity, and at the default Reynolds number for this study ($Re=2500$) the flow is fully turbulent (e.g. Bech et al. 1995). Note that this value is several orders of magnitude lower than in the atmosphere, and in Sect. 5 we discuss how this aspect may have a quantitative effect on the results.

The formal upper boundary of the system is located at $z=2h$. However, as an idealized model for the SBL the upper ‘boundary’ is located at the mid-plane $z=h$. The symmetry of the system ‘fixes’ the streamwise velocity component at $z=h$ in a statistical sense, while turbulent motions may still exist at this height. Therefore, we expect a log-linear profile up to this point. Above $z=h$ the system is symmetric with respect to the bottom part and may be advantageously used in averaging procedures by employing twice as many data points.

The choice for a fixed wind speed as upper boundary condition may appear somewhat artificial. Earlier studies investigated the collapse of turbulence, that is the transition from a weakly stable to a very stable boundary layer, using a pressure-driven (Poiseuille) flow with prescribed heat flux at the surface (Nieuwstadt 2005; Flores and Riley 2011; Donda et al. 2015b). As a more realistic model for the SBL, a Poiseuille flow appears to be the preferable candidate. Here, however, we provide motivation for the use of a Couette set-up: in real SBL flows, the transition to a VSBL is associated with decoupling of the flow from the surface (Derbyshire 1999). This causes an imbalance between the pressure force and wall friction; consequently, the flow accelerates until turbulence is restored (Businger 1973), that is the collapsed state is transient. This process is nicely captured in a Poisseuille set-up (Donda et al. 2015a). However, the time scale of this acceleration process is much larger than that of momentum redistribution (Wiel et al. 2012a), and as such, no additional momentum is generated in the early stage of the night. Here, we aim to ‘zoom in’ on this transient period after the onset of cooling, that is, by fixing total momentum the collapse of turbulence becomes permanent. This Couette set-up is more suitable for mathematical analysis as the constant-flux layer approximation is strictly valid. Moreover, a sharper transition is expected theoretically, when pressure acceleration is absent.

**Fig. 2:**
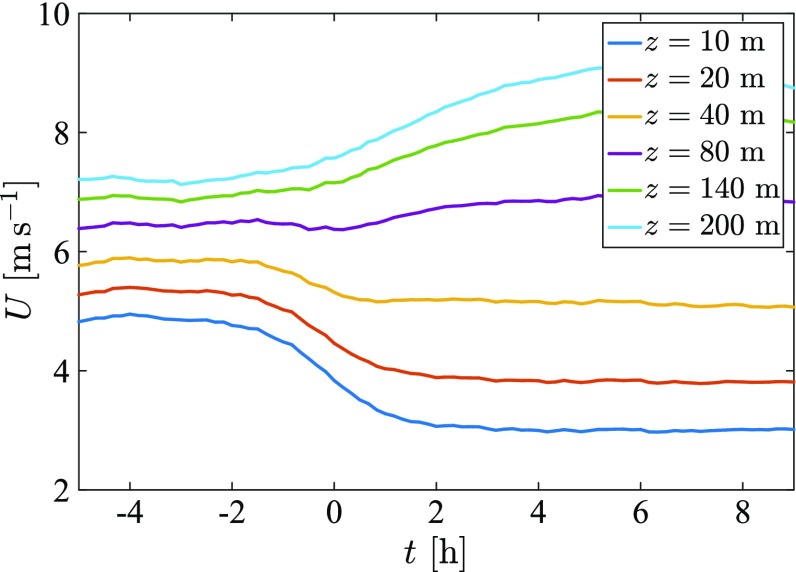
Mean wind-speed observations at the Cabauw observation tower in the Netherlands. Data are averaged over all clear-sky nights between August 2000 and December 2012. For a detailed description of this dataset, see Hooijdonk et al. (2015)). The *horizontal axis* shows the time with respect to sunset. On average, the flow accelerates after sunset for high altitudes, while at lower altitudes the flow decelerates. The crossing point appears to be in the range $z=40$–80 m. Similar results were presented in Wiel et al. (2012b)

Next, we may ask ourselves how periods with relatively constant total momentum manifest themselves in the real world. A common observation is that after sunset winds tend to weaken near the surface and flow accelerates aloft. Therefore, an altitude can be found where wind speed is relatively constant: the so-called crossing point (Wiel et al. 2012b). This observation is associated with the momentum constraint on short time scales (Wiel et al. 2012b). For example, at the Cabauw observation tower in the Netherlands, this crossing point typically occurs at between 40 and 80 m (Fig. 2). For this location, pressure acceleration typically occurs on a time scale of a 3–4 h, whereas momentum redistribution occurs within approximately 10 min. As such, at a level between 40 and 80 m the wind speed is initially (say, up to 2 h after sunset) relatively constant.

### Heat-flux boundary conditions

After sunset the SBL becomes stably stratified owing to cooling by net radiative emission. As a result, the shear production of turbulence is in competition with the buoyancy forces. For low-heat-capacity, insulated surfaces (e.g. fresh snow, short grass), surface temperatures rapidly respond to the continuous energy loss by net radiation. To mimic this effect in our model, the Couette flow is extended with heat-flux boundary conditions without explicitly modelling the energy balance of the surface itself (similar to the set-up of Nieuwstadt 2005).

Here, a downward heat flux $H_0$ is prescribed at both the top and bottom boundaries for symmetry purposes. Thus the flow is heated from the top, in addition to being cooled at the surface, such that the heat content in the system remains constant. Although $H_0$ formally refers to both cooling and heating in our model, we refer to $H_0$ as the ‘cooling rate’ or ‘surface cooling’ henceforth.

The cooling rate introduces a new dimensionless control parameter closely related to the so-called shear capacity *SC* (Hooijdonk et al. 2015). Here, this parameter is defined as

2 $$\begin{aligned} SC_C = \left[ \frac{\theta _0 \rho c_p U_0^3}{g hH_0}\right] ^{1/3}, \end{aligned}$$

where the subscript *C* refers to ‘Couette’, *g* is the acceleration due to gravity, $$\theta _0$$ is the reference temperature, $$ \rho $$ is the density and $$ c_p$$ is the specific heat capacity. This parameter compares the plate velocity $$U_0$$ to a velocity scale defined by the imposed heat flux $$H_0$$. This velocity scale is proportional to the minimum wind speed for sustained turbulence $$U_{min}$$, which is the wind speed at which $$H_0$$ is equal to the MSHF. As such, shear capacity describes whether the competition between the stabilizing buoyancy (due to cooling) and the destabilizing shear (due to plate velocity) can balance. For high $$SC_C$$ we expect a turbulent, statistically steady state, while for low $$SC_C$$ we expect that the flow will not be able to sustain the fully turbulent state. Alternatively, $$SC_C^{-3}$$ is interpreted as the dimensionless cooling rate $${\hat{H}}$$.

### Governing equations

The conservation equations (in the Boussinesq approximation) are normalized using the external parameters for this system: velocity scale $U_0$, length scale *h* and kinematic surface heat flux $H_0 / (\rho c_p)$. Combining these external parameters yields a temperature scale (subscript *scl*) with which we normalize the heat equation,

3 $$\begin{aligned} T_{scl} = \frac{H_0}{\rho c_p U_0}. \end{aligned}$$

Normalization results in the following set of dimensionless equations,

4 $$\begin{aligned} \frac{\partial u_i}{\partial x_i} = 0 \end{aligned}$$

for the conservation of mass,

5 $$\begin{aligned} \frac{\partial u_i}{\partial t} + u_j\frac{\partial u_i}{\partial x_j} = - \frac{\partial P}{\partial x_i } + \frac{1}{SC_C^{3}}T \delta _{i3} + \frac{1}{Re}\frac{\partial ^2u_i}{\partial x_j^2} \end{aligned}$$

for the conservation of momentum and

6 $$\begin{aligned} \frac{\partial T}{\partial t} + u_j\frac{\partial T}{\partial x_j} = \frac{1}{Pr Re}\frac{\partial ^2 T}{\partial x_j^2} \end{aligned}$$

for the conservation of heat (the Einstein summation convention applies). In these equations $u_i$ is the normalized velocity vector in direction *i*, with $\lbrace u_1,~u_2,~u_3\rbrace =\lbrace u,~v,~w\rbrace$, $x_i$ is the normalized position vector, with $\lbrace x_1,~x_2,~x_3\rbrace =\lbrace x,~y,~z\rbrace$, $\delta _{ij}$ is the Kronecker delta, *T* is the normalized deviation with respect to a reference temperature $\theta _0/T_{scl}$ and *P* is the normalized pressure.

The dimensionless ratio of the kinematic viscosity and heat diffusion $\kappa _\theta$ is expressed through the (molecular) Prandtl number, which is kept constant at $Pr=\nu /\kappa _\theta =1$ for simplicity. The Reynolds number is also kept constant at $Re=2500$. Observe that, besides *Re* and *Pr*, $SC_C$ appears naturally from the non-dimensionalization of the governing equations. Consequently, the behaviour of the full system is determined by these three dimensionless numbers. We investigate the effect of decreasing the shear capacity $SC_C$ (i.e. increasing ${\hat{H}}$) until a transition to the very stable state occurs.

For the analysis, the velocity and temperature fields are separated into a mean component and a fluctuating component, known as the Reynolds decomposition, that is

7a $$\begin{aligned} u =&U + u', \end{aligned}$$

7b $$\begin{aligned} v =&V + v', \end{aligned}$$

7c $$\begin{aligned} w =&W + w', \end{aligned}$$

7d $$\begin{aligned} T =&\theta + \theta ', \end{aligned}$$

where $\lbrace U,~V,~W,~\theta \rbrace$ are the horizontally-averaged fields ($V=W=0$ for all *z*), and primed quantities are the fluctuations with respect to these averaged fields.

## Solution methods

### Numerical method

The conservation equations for momentum and heat are solved using a fractional-step algorithm (Kim and Moin 1985). The implementation details are similar to the large-eddy simulation model used by Moene (2003). Here, however, the DNS configuration is used (similar to the Poiseuille set-up of Donda et al. 2015b). For time advancement a second-order accurate Adams–Bashforth technique is used; for the derivatives in space a second-order accurate finite-volume discretization is used. Output consists of vertical profiles of the first- and second-order moments (e.g. mean velocities, eddy covariances) averaged over the full horizontal domain. The domain is periodic in both horizontal directions; in the vertical direction the boundary is defined at the half-grid level. For the velocity components no-slip (Dirichlet) boundary conditions are applied, and for the heat flux Neumann boundary conditions are applied.

The domain size is $L_x \times L_y \times L_z = 10\times 10\times 2$ (normalized by the domain half-height *h*), with the number of uniformly distributed grid cells $n_x \times n_y \times n_z = 360\times 360\times 180$, where the subscripts *x*,  *y* and *z* indicate the directions. The near-neutral cases are affected by this limited domain size, in the sense that two-point correlations of $u'$ in the streamwise direction remain non-negligible over the full domain. This is a consequence of large-scale horizontal motions that exist in Couette flows (e.g. Bech et al. 1995; Komminaho et al. 1996). In our simulations this correlation becomes weaker as stability grows. The relatively small domain size causes slow temporal fluctuations, which correspond to spatial fluctuations in a larger domain (see also Deusebio et al. 2015). Similar to Tsukahara et al. (2006) and Deusebio et al. (2015), the temporally averaged first- and second-order statistics are almost insensitive to the domain size. Domain independence is verified more extensively for the neutral case and one stably stratified case by additional runs using double horizontal domains, which confirms this insensitivity (not shown).

To assess the extent to which the smallest scales of turbulence are resolved, the Kolmogorov length is estimated as (all in dimensionless units)

8 $$\begin{aligned} \eta = \left( \epsilon Re^3\right) ^{-1/4}, \end{aligned}$$

where $\epsilon$ is the dissipation rate of turbulent kinetic energy (TKE). The dissipation rate is estimated as $\epsilon \approx 0.003$ using the steady-state value of $\epsilon$ in the neutral case. Here $\epsilon$ is defined as

9 $$\begin{aligned} \epsilon =\frac{2}{Re}\left\langle \left( \frac{\partial u'_i}{\partial x_j}\right) ^2\right\rangle _D, \end{aligned}$$

where $\langle ...\rangle _D$ indicates averaging over the entire domain and summation over *i* and *j* is implied. In terms of the Kolmogorov length, the grid resolution for the neutral case is estimated as $\Delta x \times \Delta y \times \Delta z = 2.3\eta \times 2.3\eta \times 0.9 \eta$ (for stably stratified cases, the resolution increases; see next section). As such, we are at the limit of fully resolving the Kolmogorov scale. However, as discussed in Nieuwstadt (2005) and Donda et al. (2015b), we expect the effects of static stability to manifest themselves primarily at the larger scales. Also, Moin and Mahesh (1998) suggest that DNS can still achieve acceptable accuracy using grid resolutions slightly larger than $\eta$ in the horizontal direction. We verified that simulations with the default resolution and with double horizontal resolution yield close agreement with the logarithmic-law diagnostic function as presented in Pirozzoli et al. (2014) (not shown).

The present set-up and code must be validated; therefore, the consistency of the current results with the literature (Tsukahara et al. 2006) and analytical solutions is assessed in Sect. 3.3.

**Table 1:** Overview of configuration for each run. The columns read (left to right) as follows: run label, Reynolds number, simulation length, resolution in wall units $\Delta _i^+$, shear capacity, dimensionless surface cooling, domain size and field used as initial condition (IC)

Run	*Re*	$t [h/U_0]$	$\Delta ^+_x$	$\Delta ^+_y$	$\Delta ^+_z$	$SC_C$	${\hat{H}} [\cdot 10^{-5}]$	$[L_x,~L_y,~L_z]$	IC
T1	750	300	4.4	1.2	1.0	Inf	0	[44.8, 6, 1]	–
T2	2150	300	12.2	6.1	3.6	Inf	0	[24, 6, 1]	–
N	2500	600	4.0	4.0	1.6	Inf	0	[10, 10, 2]	–
S1	2500	600	3.9	3.9	1.6	79.4	0.2	[10, 10, 2]	N
S2	2500	600	3.9	3.9	1.6	63.0	0.4	[10, 10, 2]	N
S3	2500	600	3.9	1.6	3.9	55.0	0.6	[10, 10, 2]	N
S4	2500	600	3.9	1.6	3.9	46.4	1	[10, 10, 2]	N
S5	2500	600	3.9	1.6	3.9	40.5	1.5	[10, 10, 2]	S4
S6	2500	600	3.8	1.5	3.8	36.8	2	[10, 10, 2]	S5
S7	2500	600	3.7	3.7	1.5	32.2	3	[10, 10, 2]	S6
S8	2500	600	3.7	3.7	1.5	29.2	4	[10, 10, 2]	S7
S9	2500	600	3.6	3.6	1.4	27.1	5	[10, 10, 2]	S8
S10	2500	600	3.5	3.5	1.4	25.5	6	[10, 10, 2]	S9
S11	2500	600	3.3	3.3	1.3	24.3	7	[10, 10, 2]	S10
S12	2500	600	3.1	3.1	1.2	23.7	7.5	[10, 10, 2]	S11
S13	2500	600	3.1	3.1	1.2	23.4	7.75	[10, 10, 2]	S12
S14*	2500	1200	2.5	2.5	1.0	23.2	8	[10, 10, 2]	S13
S15*	2500	600	2.5	2.5	1.0	22.7	8.5	[10, 10, 2]	S13
S16*	2500	450	2.5	2.5	1.0	22.3	9	[10, 10, 2]	S13
S7-N	2500	600	3.7	3.7	1.5	32.2	3	[10, 10, 2]	N
S9-N	2500	600	3.6	3.6	1.4	27.1	5	[10, 10, 2]	N
S10-N	2500	600	3.5	3.5	1.4	25.5	6	[10, 10, 2]	N
S11-N	2500	600	3.3	3.3	1.3	24.3	7	[10, 10, 2]	N

The number of grid points is as follows: for run T1 $$[n_x,~n_y,~n_z]= [1024,~512,~96] $$; for T2 $$[n_x,~n_y,~n_z]= [512,~256,~72]$$; and all other runs $$[n_x,~n_y,~n_z]= [360,~360,~180] $$. Runs marked by an asterisk do not become steady within the simulated time

### Strategy

Table 1 shows an overview of the investigated configurations, and to initialize the neutral case ($SC_C\rightarrow \infty$), an artificial flow field is used. This field consists of two super-positioned parts: a horizontally homogeneous logarithmic profile, which is predicted analytically, and an inhomogeneous flow field to initialize turbulence, which consists of randomly oriented harmonic oscillations of different phases and wavelengths. The flow field is allowed to develop until $t = 100 h/U_0$ to obtain a fully turbulent field that is uncorrelated with the initialization. Runs T1, T2 and N (Table 1) are started from such turbulent fields.

The comparison runs T1 and T2 use configurations as in Tsukahara et al. (2006). Note that our definition of the Reynolds number is used, which is different from that in Tsukahara et al. (2006). Run N provides the neutrally stratified reference case, which is continued long enough that a statistically steady state exists. Next, the buoyancy term in Eq. 5 is systematically increased by decreasing $SC_C$ (i.e. increasing ${\hat{H}}$). It is expected that if ${\hat{H}}>{\hat{H}}_\mathrm{crit}$, no statistically steady turbulent state can be found, and that the turbulent intensity suddenly decreases to much smaller levels. We investigate the value of ${\hat{H}}_\mathrm{crit}$ for this particular set-up and how the steady state of the flow changes in the case ${\hat{H}}<{\hat{H}}_\mathrm{crit}$.

For each run the statistically steady state, which results from a prior run (one step less stable), is used as a starting point. For runs S1–S4 this starting point is provided by run N, whereas runs S5–S14 are initialized with the statistically steady state of runs S4–S13, respectively. Especially close to the critical point this stepwise approach is necessary since large steps may result in the collapse of turbulence, despite the fact that a turbulent steady state still exists. Runs S15 and S16 are both initialized with run S13 because runs S14 and S15 do not reach a statistically steady state. Four additional runs (S7-N, S9-N, S10-N and S11-N) are performed to investigate the typical time needed to reach a steady state, as well as to verify that the results are independent of the initial conditions. These runs use the same cooling rates as runs S7, S9, S10 and S11 but are initialized with neutral conditions (i.e. run N).

The resolution in terms of wall units is defined as

10 $$\begin{aligned} \Delta _i^+ = \Delta _i Re_\tau , \end{aligned}$$

where the subscript *i* denotes the directions (*x*, *y* or *z*), and $Re_\tau =u_*h/\nu$ ranges from $Re_\tau \approx 145$ for the neutral case to $Re_\tau \approx 110$ for run S13. The friction velocity $u_*$ is diagnosed from the DNS results,

11 $$\begin{aligned} u_*^2 = \frac{1}{Re}\langle (\partial U/\partial z)_{z=0}\rangle _{t}, \end{aligned}$$

where $\langle ...\rangle _{t}$ denotes additional averaging over time.

The simulation length of each run is $t = 600~h/U_0$, and to indicate how the simulation length corresponds to dimensional time, the values from Fig. 2 are taken as an example, that is $U_0\approx 5~\hbox {m} \, \hbox {s}^{-1}$ and $h\approx 40~\hbox {m}$. Using these values, we find that a simulation length of $600~h/U_0$ corresponds to 1.5 h of dimensional time in a typical NBL. Since a strongly idealized system is used here, this value should be taken as an order-of-magnitude estimate only.

### Validation

Couette flows have been studied extensively for the neutral, non-stratified case. For example, Tsukahara et al. (2006) published an overview of several numerical and experimental studies of this type of flow for various configurations (domain sizes, Reynolds numbers). Two of their runs were repeated using the current code (T1 and T2) to assess the performance of the current numerical method with respect to their benchmark. We define these cases using the Reynolds number based on the domain half-height, rather than the full domain height as in Tsukahara et al. (2006).

Stably stratified Couette flows have been limited to configurations which use a fixed temperature as a boundary condition. Therefore, MO similarity profiles are used for comparison with the stably stratified cases.

As Tsukahara et al. (2006) presented their results in wall units, we adopt the same normalization,

12 $$\begin{aligned} u'^+_{i} = \frac{\langle u_i'^2\rangle ^{1/2}_{H,t}}{u_*}, \end{aligned}$$

where $\langle ... \rangle _H$ denotes averaging over the full horizontal plane and *i* denotes the velocity component (*u*,  *v* or *w*). Note that $u'_i$ and $u_*$ are already normalized using $U_0$. The vertical position in wall units reads (all dimensionless),

13 $$\begin{aligned} z^+ = z~Re_\tau . \end{aligned}$$

**Fig. 3:**
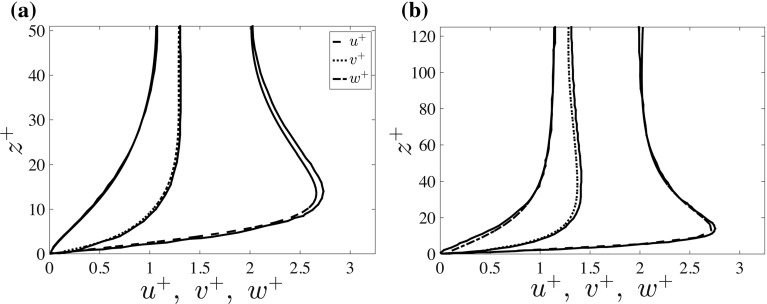
Comparison of dimensionless velocity fluctuation profile with Tsukahara et al. (2006) for cases T1 (**a**) and T2 (**b**). *Solid lines* are obtained from Tsukahara et al. (2006) with a data digitiser. *Dashed lines* represent root-mean-squared fluctuations of the three velocity components (Eq. 12) as indicated in the legend

Although we did not perform a formal statistical error analysis, the close agreement in Fig. 3 suggests that our simulations are able to reproduce the results of Tsukahara et al. (2006).

**Fig. 4:**
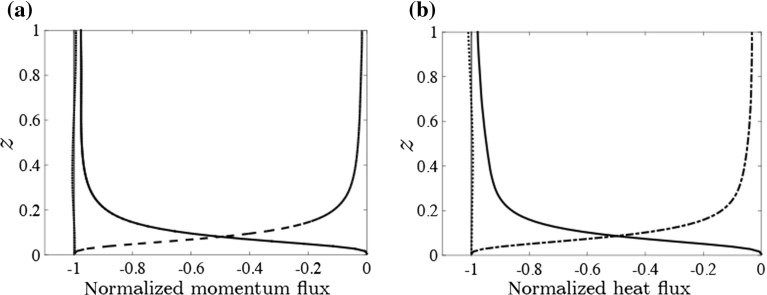
Momentum (**a**) and heat (**b**) fluxes normalized using $u_*$ for case S8 ($SC_C=29$): turbulent fluxes (*solid*), diffusive fluxes (*dash-dot*) and total flux (*dotted*). The *thin vertical line* indicates the predicted total flux based on its surface value

Figure 4a, b shows profiles of the normalized turbulent and viscous fluxes of momentum and heat for a stably stratified case ($SC_C = 29,~{\hat{H}}=4\times 10^{-5}$) and verifies that the total heat flux is equal to the imposed heat flux over the full height. Furthermore, it shows that the turbulent fluxes of both heat and momentum are relatively constant in the centre of the flow.

In general, the governing equations cannot be solved analytically. Under certain assumptions, however, mean velocity and temperature profiles can be obtained. For brevity we restrict ourselves to introducing the main assumptions, while referring the reader to Wiel et al. (2007) for a full derivation.

We assume that the flow is statistically steady and horizontally homogeneous. Since fluxes are independent of the height, we additionally assume that the mean profiles can be described with MO theory (Monin and Obukhov 1954) and the Businger–Dyer flux-profile relations (Businger et al. 1971), with the turbulent Prandtl number $Pr_T=1$.

The dimensionless equations for momentum and heat then reduce to

14 $$\begin{aligned} 0 = \frac{\partial U}{\partial t} = \frac{\partial \tau }{\partial z} = \frac{\partial }{\partial z}\left( (\kappa z)^2\left( \frac{\partial U}{\partial z} \right) ^2(1 - \alpha R_i)^2\right) \end{aligned}$$

and

15 $$\begin{aligned} 0 = \frac{\partial \theta }{\partial t} = -\frac{\partial H}{\partial z} = \frac{\partial }{\partial z}\left( (\kappa z)^2\frac{\partial \theta }{\partial z}\frac{\partial U}{\partial z}(1-\alpha R_i)^2\right) . \end{aligned}$$

Here, $\alpha =4.5$ is obtained from our DNS results and $\kappa =0.4$ is the von Kármán constant (Hogstrom 1996). The value for $\alpha$ appears to fit within the range found in the literature (e.g. Howell and Sun 1999; Wiel et al. 2008; Ansorge and Mellado 2014). The Richardson number is defined as (using non-dimensionalized gradients)

16 $$\begin{aligned} R_i = SC_C^{-3}\frac{\partial \theta }{\partial z}\left( \frac{\partial U}{\partial z}\right) ^{-2}. \end{aligned}$$

**Fig. 5:**
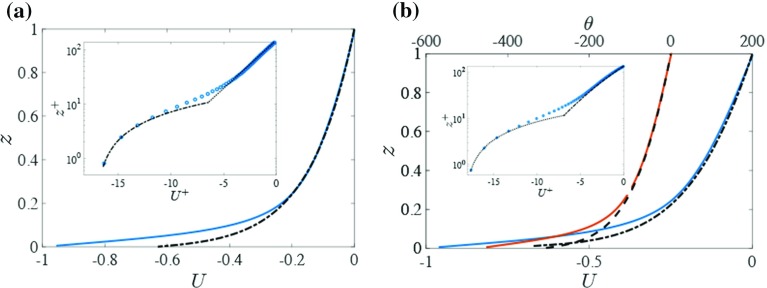
Comparison between (dimensionless) theoretical profiles for (**a**) case N (neutral), and (**b**) case S8 ($SC_C=29$). *Blue line*: simulated velocity profiles; *black dash-dot*: log-linear velocity profile (obtained from Eqs. 14 and 15); *orange line*: simulated temperature profile; *dashed line*: log-linear temperature profile (Eqs. 14 and 15). *Insets* show the same comparison of velocity profiles in wall units, with the addition of the line $U^+=z^+ - U_0^+$ (*black dotted*), indicating linear near-wall behaviour

Velocity and temperature profiles, as well as turbulent fluxes, can be obtained from these equations for the weakly stable state. The velocity profiles for two cases (neutral and $SC_C = 29$) are compared to the profiles obtained from Eqs. (14)–(16) (Fig. 5). General agreement with MO similarity is good, except in the buffer layer, where MO similarity becomes invalid.

The agreement of the mean flow properties between our results and the benchmark cases (Tsukahara et al. 2006 and MO similarity) indicates that our results are plausible for both cooled and neutral system configurations. Additionally, Fig. 4a, b shows that the diffusive transport of heat and momentum is limited to a few per cent in the centre of the flow.

**Fig. 6:**
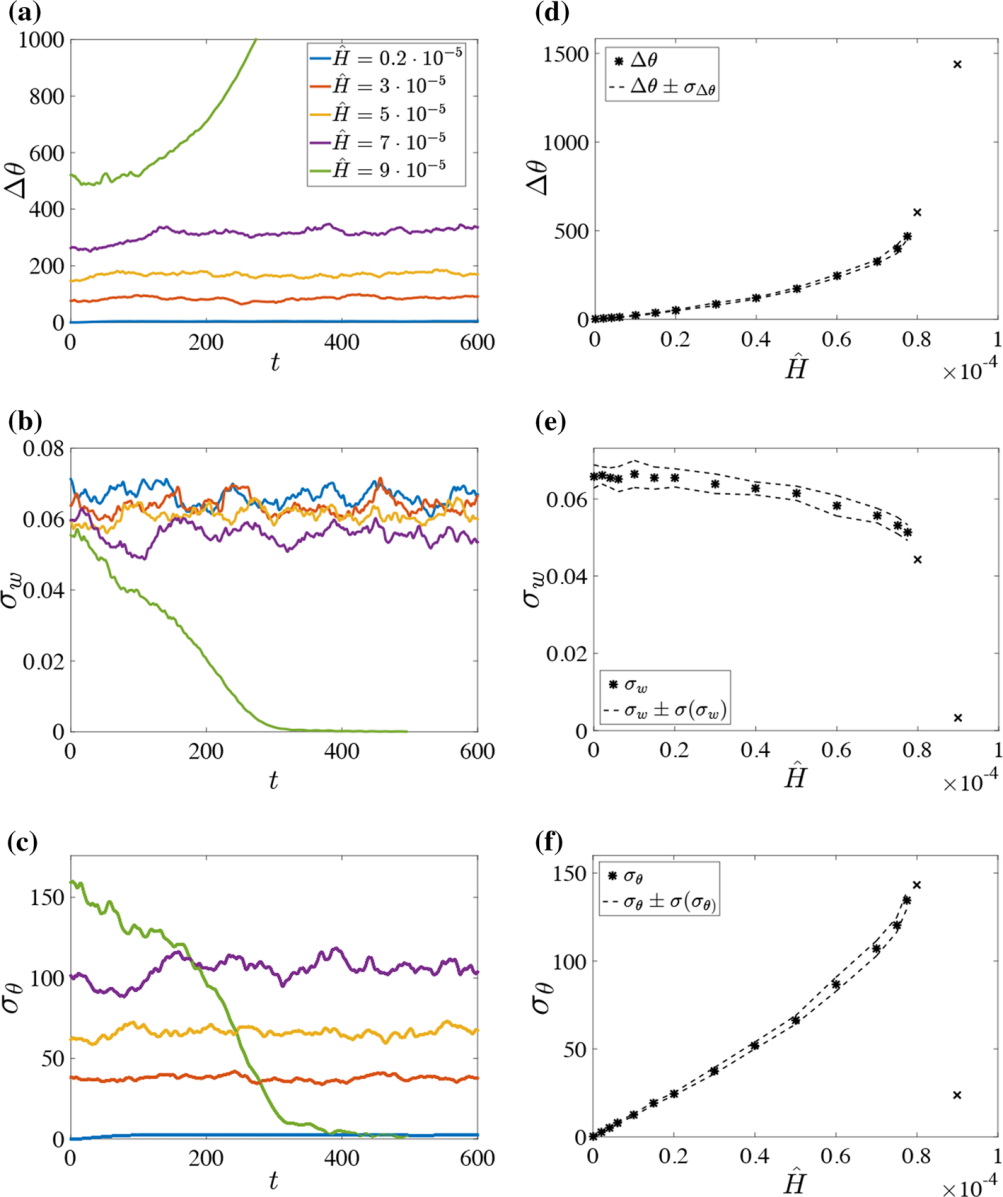
(*Left column*) Temporal evolution of **a** temperature difference $\Delta \theta$, **b** mean vertical velocity fluctuation and **c** mean temperature fluctuation (all dimensionless). Averages are between $z=0.5$ and $z=-1.5$ and over the full horizontal domain. *Colours* are as indicated in the legend. *Right column*: Steady-state values of **d** $\Delta \theta$, **e** $\sigma _w$ and **f** $\sigma _{\theta }$ as a function of the dimensionless cooling rate. For a cooling rate ${\hat{H}}> 8.5\times 10^{-5}$ no turbulence can be sustained, while the temperature difference increases sharply. The *dashed lines* indicate the standard deviation as defined in the main text. These *lines* may be interpreted as *error bars*. Note that the *crosses* indicate that no equilibrium has been reached. For these data points the flow likely laminarizes eventually

## Results

### Temporal evolution

Here, the temporal behaviour for all cases (N, S1–16, Table 1) is investigated. Figure 6a–c shows typical results (intermediate cases are omitted for clarity). These results are obtained in the centre of the flow domain ($0.5< z < 1.5$) since this region remains largely unaffected by viscous effects. As a proxy for turbulence intensity, the vertical velocity fluctuations $\sigma _w$ are used, defined as

17 $$\begin{aligned} \sigma _w = \langle w'^2\rangle _{C}^{1/2}, \end{aligned}$$

where $\langle ...\rangle _{C}$ denotes averaging over the full horizontal domain and over the centre region ($0.5<z<1.5$). Note that we exploit the symmetry of the system to collect the statistics from the bottom half as well as the top half of the domain.

We prefer the fluctuations of the vertical velocity component as a measure of the turbulence intensity over, for example, the TKE (cf. Shah and Bou-Zeid 2014). The TKE may be affected by large-scale horizontal meandering motions (e.g. Bech et al. 1995). These motions result in increased variance with respect to the mean flow for the horizontal components, but they should not be seen as three-dimensional turbulence (e.g. Hanna 1983; Hutchins and Marusic 2007; Mahrt et al. 2009).

The temperature fluctuations $\sigma _{\theta }$ are defined analogously to $\sigma _w$, and we also define a characteristic measure for the inversion strength by

18 $$\begin{aligned} \Delta \theta = \theta (z=1.5)-\theta (z=0.5). \end{aligned}$$

Figure 6a–c shows that the weakly stable cases (${\hat{H}} \lesssim 8\times 10^{-5}$) reach a (statistically) steady state after some adjustment period (defined as $t<200~h/U_0$). Interestingly, the response of the vertical velocity fluctuations $\sigma _w$ to increased cooling appears quite weak for these cases. Case S16 (green), however, is clearly distinct from the other cases, as shown by the sharp increase in $\Delta \theta$ (Fig. 6a) and the sharp decrease in fluctuations $\sigma _w,\sigma _{\theta }$ (Fig. 6b, c). This qualitative transition may be explained by MSHF theory, that is for supercritical surface cooling the flow becomes very stable and turbulence is strongly suppressed.

A closer inspection of intermediate cases (not shown) indicates that the critical cooling ${\hat{H}}_\mathrm{crit}$ occurs in the interval ${\hat{H}}\in [8.0\times 10^{-5};8.5\times 10^{-5}]$, corresponding to $SC_C = [23.2;22.7]$. Within this range, turbulence decays slowly and is never stationary. An extension (up to $t=1200~h/U_0$) of run S14 (${\hat{H}}=8\times 10^{-5}$) verified that the decay continues and that turbulence becomes very weak (i.e. less than 1 % of the initial intensity).

### Atmospheric example

Owing to the normalization, it is not straightforward to interpret these results in the atmospheric context. To facilitate a more direct interpretation, typical values for atmospheric flows are used to present some results in dimensional form: air density $\rho =1.2$ kg m$^{-3}$, specific heat capacity $c_p=1005 \, \hbox {J} \, \hbox {K}^{-1}\, \hbox {kg}^{-1}$, typical air temperature $\theta _0=285$ K and $g=9.81 \, \hbox {m} \, \hbox {s}^{-2}$. Again we take $U_0=5$ m  s$^{-1}$ and $h=40$ m. The *dimensional* cooling rate can be determined by

19 $$\begin{aligned} H_0 = \frac{U_0^3\theta _0\rho c_p}{gh}{\hat{H}}, \end{aligned}$$

and since ${\hat{H}}$ varies from zero to $9\times 10^{-5}$, the dimensional equivalent $H_0$ varies from zero to 10 Wm$^{-2}$. As such, the transition to a VSBL occurs roughly when the cooling rate exceeds 9 Wm$^{-2}$. The temperature difference (measured between the surface and the centre of the flow) is approximately constant around a value of 985 when ${\hat{H}}=7\times 10^{-5}$ (purple line). This corresponds to a dimensional equivalent temperature difference of 1.3 K over 40 m. Considering the strong idealization in our set-up, these values seem reasonable as a rough estimate for the real NBL (e.g. as compared to Hooijdonk et al. 2015, where the temperature difference is approximately 2 K over 40 m) . Also, the critical value $SC_C\approx 23$ (as found in Sect. 4.1) corresponds closely ($\lesssim 5$ % difference) to the value found in Hooijdonk et al. (2015) (using their Eq. 7).

### Steady state

From Fig. 6a–c it becomes clear that the (statistically) steady state is affected by the increased surface cooling. Figure 6d–f depicts the response of the steady state, defined by the time-averaged ($200<t<600~h/U_0$) value of $\lbrace \Delta \theta , ~\sigma _w,~ \sigma _{\theta } \rbrace$, to the increased surface cooling, with the magnitude of the temporal fluctuations during the steady period (Fig. 6a–c) interpreted as ‘error bars’ in Fig. 6d–f (dashed lines). This magnitude is defined as

20 $$\begin{aligned} \sigma (\sigma _w) = \langle (\sigma _w-\langle \sigma _w\rangle _t)^2\rangle _t^{1/2}, \end{aligned}$$

where $\langle ...\rangle _t$ indicates averaging over time. Equivalent definitions apply to $\sigma (\Delta \theta )$ and $\sigma (\sigma _{\theta })$.

Figure 6d shows how the temperature difference is affected by the surface cooling. The effect of the increased cooling becomes more pronounced as the critical point is approached, where the increased effect is explained using MSHF theory in the next section.

Figure 6e shows that $\sigma _w$ is not as strongly affected as $\Delta \theta$. Also, the effect of the increased cooling is weak compared to the fluctuations $\sigma (\sigma _w)$, except close to the critical point. This weak response may be expected, since at the point of collapse the bulk Richardson number as measured between $z=0$ and $z=h$ is still relatively small, that is $R_b =SC_C^{-3} h[\theta (h)-\theta (z_0)] / U_0^2 = 0.3$. This is close to the value predicted based on MO similarity (Wiel et al. 2007, $R_{i}\le 1/(3\alpha )\approx 0.07$, with $\alpha =4.5$ in our case).

The temperature fluctuations are shown in Fig. 6f, and observe that when ${\hat{H}}<{\hat{H}}_\mathrm{crit}$, the temperature fluctuations increase with increasing ${\hat{H}}$. Thus, it appears that the decrease in vertical motion $\sigma _w$ that would lead to a reduced temperature variance is compensated by an increased temperature gradient. Conversely, in the case where ${\hat{H}}>{\hat{H}}_\mathrm{crit}$, the temperature fluctuations become very small owing to the absence of velocity fluctuations. Similar to $\Delta \theta$ and $\sigma _w$, the slope of $\sigma _{\theta }$ with respect to ${\hat{H}}$ appears to increase prior to collapse.

### Early warning signals for critical transitions

The regime transition appears to be preceded by an increased slope in Fig. 6d–f. Hence, we explore whether the turbulent flows contain ‘hidden’ information (e.g. change in slope) about a nearing collapse. Following the nomenclature on transitions in generic non-linear dynamical systems (e.g. Scheffer et al. 2009), we refer to this information as ‘early warning signals’.

**Fig. 7:**
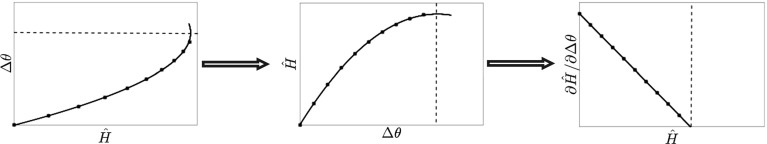
Sketch of employed strategy. The *axes* of the *left* figure are swapped to arrive at the *centre* figure. The slope of these (virtual) data points is determined and plotted in the figure on the *right*. These data points are interpolated with a linear fit (*solid line*) and used to predict the transition point (*dashed line*)

A quantitative estimate for the critical point could also be obtained if one adopted a specific turbulence closure like MO similarity (e.g. Wiel et al. 2007; Donda et al. 2015b). However, we aim to make such a prediction *independent* of any closure here by using the MSHF theory. Note that, qualitatively, the existence of an MSHF can be explained without relying on a closure model (Sect. 1) and as such, an increased slope is predicted qualitatively for $\Delta \theta$ (Fig. 7). Therefore, we first use $\Delta \theta$ to infer the critical cooling rate; later, the slopes of $\sigma _w$ and $\sigma _{\theta }$ are also investigated.

MSHF theory is used to infer the critical point in the following manner: to have a turbulent steady state, we require $H_\mathrm{act} = {\hat{H}}$, that is the actual turbulent heat transport should be able to compensate the energy loss at the surface. MSHF theory explains that the actual heat flux is limited to a certain maximum. At the maximum, an intensification of the temperature gradient does not result in more downward heat transport because the vertical mixing is strongly suppressed. This maximum occurs in the case where

21 $$\begin{aligned} 0=\frac{\partial H_\mathrm{act}}{\partial \Delta \theta } =\frac{\partial {\hat{H}}}{\partial \Delta \theta }, \end{aligned}$$

and at this point, heat transport $H_\mathrm{act}$ cannot become larger, irrespective of $\Delta \theta$. Conversely, a slight increase in ${\hat{H}}$ causes an ‘infinite’ increase in $\Delta \theta$ (though formally the increase is limited by viscous heat transport). These equivalent statements are illustrated in Fig. 7; the current results are sketched in the left panel and in the centre panel, the axes are exchanged, such that MSHF theory is illustrated. Finally, in the right panel, the slope $\partial {\hat{H}}/\partial \Delta \theta$ is sketched as a function of ${\hat{H}}$. These data points are interpolated with a linear fit and used to estimate the critical point. The strategy as sketched in Fig. 7 is applied to interpret our results, and because a limited number of data points are available, a finite difference approximation is used to obtain the slope.

**Fig. 8:**
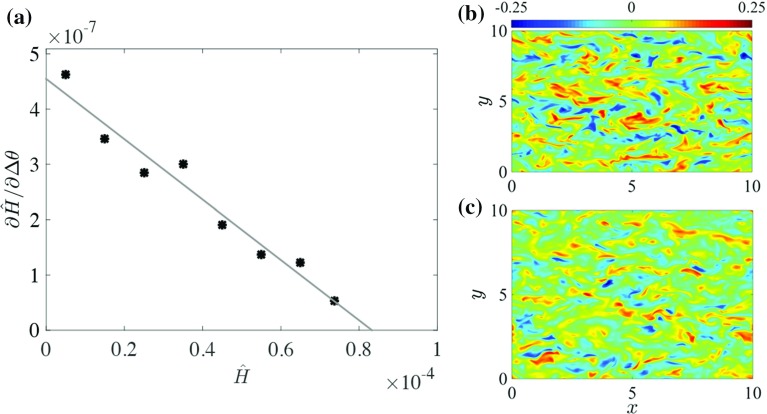
**a** Inverted response of $\Delta \theta$ to increased cooling as function of cooling rate. *Black asterisks* represent finite difference approximations to $\partial {\hat{H}}/ \partial \Delta \theta$. Only data points with approximately equal steps in ${\hat{H}}$ are used. The *thin grey line* represents a linear fit through the data points. **b** Snapshot of vertical velocity field at $z=1$ for case $SC_C=36.8$ (${\hat{H}}=2\times 10^{-5}$). **c** Same as **b** for case $SC_C=24.3$ (${\hat{H}}=7\times 10^{-5}$)

With respect to $\Delta \theta$, the results of the foregoing strategy are depicted in Fig. 8a where it appears that the tendency towards a maximum, that is zero slope, is almost linear. The quality of the fit is confirmed by a value $R^2\approx 0.95$ for a linear fit. By extrapolating the data points linearly to the horizontal axis, a prediction for the critical point ${\hat{H}}_{crit}\approx 8.5\times 10^{-5}$ is obtained, which is close to the observed critical cooling rate (e.g. in Fig. 6d–f). The cross-sections in Fig. 8b, c indicate that close to the transition point, no apparent signs of an imminent collapse are present. This can be explained by the relatively low stability at which this type of collapse occurs (cf. intermittent case in Ansorge and Mellado 2014).

**Fig. 9:**
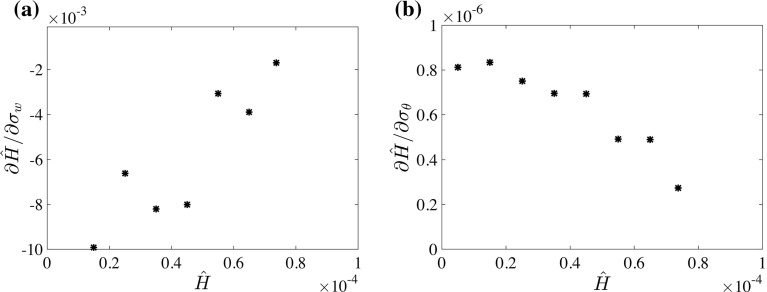
Inverted response of $\sigma _w$ (**a**) and $\sigma _{\theta }$ (**b**) to increased cooling as a function of surface cooling. *Black asterisks* represent finite difference approximations to $\partial {\hat{H}}/\partial (\Delta \theta )$

From Fig. 6e, f it also appears that the magnitude of the slope increases for $\sigma _w$ and $\sigma _{\theta }$, when the system approaches the critical point. The same procedure as for $\Delta \theta$ is used to estimate the critical point using $\sigma _w$ and $\sigma _{\theta }$. In Fig. 9a, b the slopes with respect to $\sigma _w$ and $\sigma _{\theta }$ show quite irregular behaviour when weak surface cooling is applied. Close to neutral conditions, the slope is mainly determined by the natural fluctuations in the turbulence intensity, rather than by the response to the increased surface cooling (cf. Fig. 6). This irregularity is probably a consequence of limited statistical convergence, and a larger domain size may provide a solution (García-Villalba and Álamo 2011). However, when surface cooling approaches the critical cooling rate, Fig. 9a, b suggests that an extrapolation of the data would cross the horizontal axis below ${\hat{H}}=1\times 10^{-4}$. Thus, tentatively, it appears that $\sigma _w$ and $\sigma _{\theta }$ can be used to obtain a rough estimate of the critical point.

### Generic early warning signals

MSHF theory is specific to our physical system. For general non-linear systems other signals exist that precede a critical transition in general. Scheffer et al. (2009) identify a so-called critical slowing down as such a generic marker, where slowing down refers to the observation that dynamic systems tend to recover from perturbations on longer time scales when the system is closer to a critical point (see Appendix for a more elaborate introduction). Scheffer et al. (2012) reviewed critical transitions in a wide variety of fields ranging from climate systems to financial systems: a common prerequisite for critical transitions appears to be the presence of a positive feedback, which propels the system to an alternative state (in our case: the laminar state) once a certain threshold is passed (Angeli et al. 2004; Scheffer et al. 2012). In the cooled Couette flow, such a positive feedback mechanism between decreased turbulent heat flux and increased temperature gradient is present (Sect. 2.1, Wiel et al. 2007). A more realistic model indicates that this behaviour may also exist in the real NBL (McNider et al. 1995). As such, we investigate whether indicators for critical slowing down can be observed in this system as well.

The occurrence of slowing down is tested by measuring the typical rate (defined below) at which a statistically steady state is approached. This rate is obtained in simulations that use the neutrally stratified case (run N) as the initial condition. This time can be determined for simulations S1–4. To extend the dataset, additional runs S7-N, S9-N, S10-N and S11-N are performed.

**Fig. 10:**
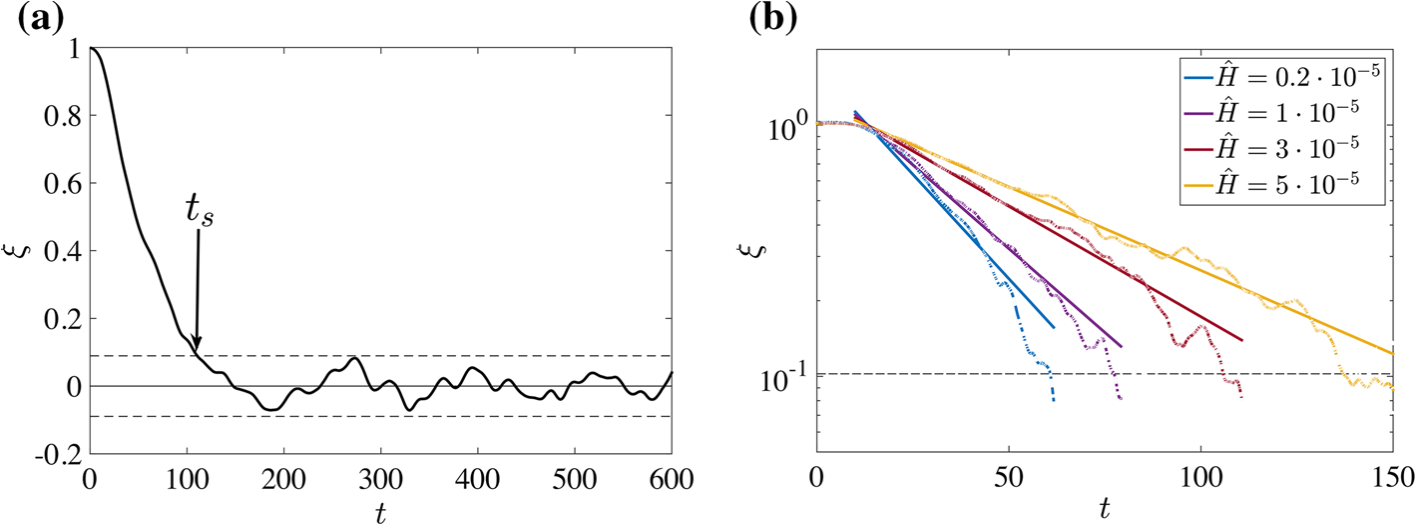
**a** Distance $\xi$ to equilibrium as function of time for a single case and **b** on a semi-logarithmic scale for a few cases. *Dashed horizontal lines* indicate the magnitude of the typical turbulent fluctuations $\sigma _{\Delta \theta }$. The time $t_s$ is defined as the first time $\xi < \sigma _{\Delta \theta }$. *Thin solid lines* in **b** are exponential fits according to Eq. (23)

First, we define the magnitude of the perturbation,

22 $$\begin{aligned} \xi (t) = \frac{\Delta \theta (t) - \langle \Delta \theta \rangle _t}{\Delta \theta (t=0) - \langle \Delta \theta \rangle _t}, \end{aligned}$$

as the relative ‘distance’ to equilibrium as a function of time. Here, $\langle ...\rangle _t$ denotes the time-averaged steady state as obtained previously (cf. Fig. 6d). As an illustration, Fig. 10a shows $\xi (t)$ for a single run (S7-N).

The relaxation stage is defined as the period between $t=0$ and $t=t_s$. The end of this stage $t_s$ is defined as the time when $\xi$ becomes smaller than the typical magnitude of the turbulent fluctuations $\sigma _{\Delta \theta }$, as illustrated in Fig. 10a. Between $t=0$ and $t=t_s$ the time series is fitted with an exponentially decaying function,

23 $$\begin{aligned} y = Ae^{-t/\tau }, \end{aligned}$$

where $\tau$ is taken as the typical relaxation time, that is the time scale of approach to equilibrium. Figure 10 suggests that the exponential fit is a reasonable choice as a proof of principle, and in Fig. 10b $\xi$ is shown on semi-logarithmic axes, fitted with Eq. 23. Runs S2, S3, S10-N and S11-N are omitted from this figure for clarity. The slope in Fig. 10b is a measure of the time constant $\tau$.

**Fig. 11:**
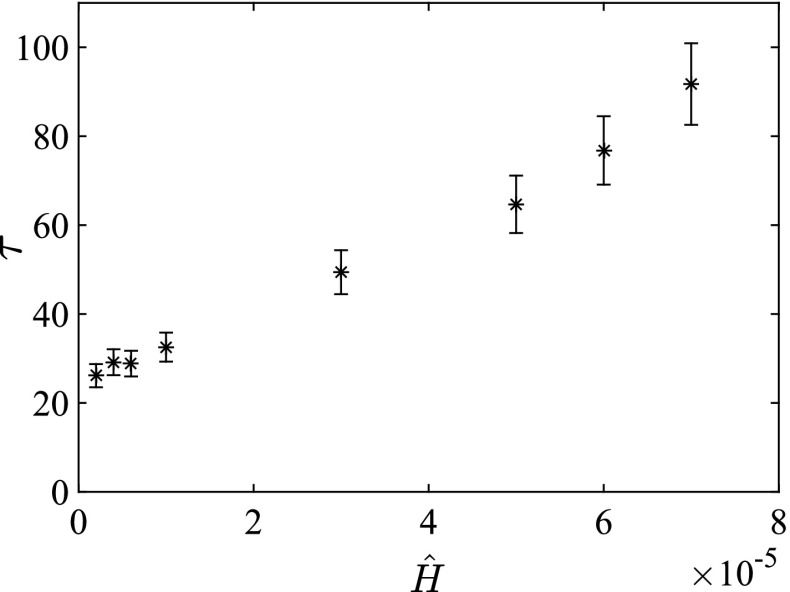
Typical time scale (as defined in text) to reach equilibrium state. *Error bars* are obtained from separate low-resolution runs, as explained in the main text

Figure 11 shows $\tau$ as a function of the surface cooling. Indeed, the relaxation time increases when the critical point is approached. If we consider an alternative time scale such as $h/\sigma _w$, we find that it cannot explain the increase in $\tau$; cf. the relaxation towards the laminar state in Flores and Riley 2011). For ${\hat{H}}=1\times 10^{-5}$ ($SC_C=46.4$, run S4) and ${\hat{H}}=3\times 10^{-5}$ ($SC_C=32.2$, run S7), the runs are repeated several times using varying realizations of the neutral state as an initial field. To limit computational costs, these runs are performed at a lower resolution. The relative spread in $\tau$ from these low-resolution runs is used to estimate the size of the error bars as presented in Fig. 11.

In the well-controlled environment of this study, the relaxation time can be measured directly. By contrast, for real situations, for example ocean or atmospheric systems, it is often not feasible to apply well-controlled perturbations. Additionally, large spontaneous perturbations may occur naturally. For such situations, alternative, indirect indicators may be used (e.g. Wissel 1984; Scheffer et al. 2001; Lenton et al. 2012; Wang et al. 2012), and here we use the relative magnitude of the standard deviation as such an indicator, defined as

24 $$\begin{aligned} {\hat{\sigma }}(\Delta \theta ) = \frac{\sigma (\Delta \theta )}{\langle \Delta \theta \rangle _t}. \end{aligned}$$

**Fig. 12:**
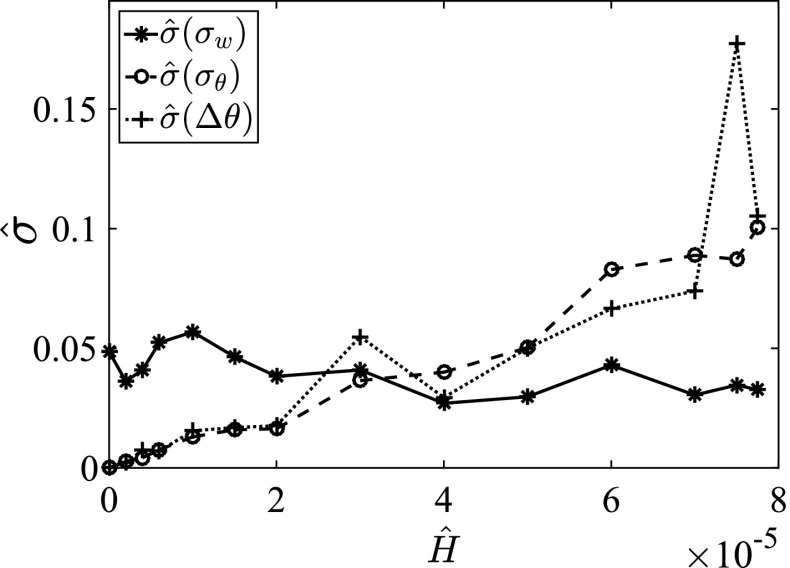
Normalized fluctuations with respect to time-averaged values between $t=200$ and $t=600~h/U_0$ for $\Delta \theta$ (*plus-signs*), $\sigma _w$ (*asterisks*) and $\sigma _{\theta }$ (*circles*). Note that the peak in ${\hat{\sigma }}(\Delta \theta )$ at ${\hat{H}}=7.5\times 10^{-5}$ does not appear if other averaging periods are used. As such, it appears to be an outlier due to limited statistical convergence

Equivalent definitions apply to $\sigma (\sigma _w)$ and $\sigma (\sigma _{\theta })$. When slowing down becomes apparent, perturbations become more persistent, that is the relaxation time increases. As such, we expect that the normalized standard deviation will increase when the system approaches the critical point. Figure 12 indicates that this is the case for ${\hat{\sigma }}(\Delta \theta )$ and ${\hat{\sigma }}(\sigma _{\theta })$. However, this increase in itself is insufficient to prove that slowing down occurs (Neubert and Caswell 1997; Dakos et al. 2012). Moreover, the fluctuations in $\sigma _w$ do not show such an increase. A possible explanation for the absence of such an increase in ${\hat{\sigma }}(\sigma _w)$ is that fluctuations of the turbulent intensity occur naturally, and these may not be easily distinguished from wave activity. Additionally, long-term fluctuations may occur in the small-size system used here (Deusebio et al. 2015).

## Discussion

A Couette flow with heat-flux boundary conditions was used to study the dynamical behaviour of the stable boundary layer in an idealized setting. The dimensionless ratio of surface cooling and wind speed is expressed through the parameter $SC_C$. This ratio is determined based on dimensional arguments only and is strongly related to the shear capacity *SC* as defined in Hooijdonk et al. (2015), where closure-dependent terms are included in the definition, such that $SC\approx 1$ is expected as a critical point.

Using field observations, Hooijdonk et al. (2015) and, later, Monahan et al. (2015) found that if the shear capacity is less than a critical value, then the WSBL undergoes a transition to the VSBL. This transition occurs globally as the system is propelled to higher stability levels by a positive feedback between stronger stratification and a weaker turbulent heat flux. Our results do not indicate what happens locally during the transition, for example the possible co-existence of turbulent–laminar spots. Such phenomena appear to be controlled by the scale separation between the Obukhov length and the wall unit $h/u_*$ ($Re_L$ and $L_+$ in Flores and Riley 2011; Deusebio et al. 2015).

As with earlier studies (e.g. Shah and Bou-Zeid 2014; Ansorge and Mellado 2014; Deusebio et al. 2015), close agreement between DNS results and MO similarity is found within the WSBL. This suggests that DNS results may be extended to the real SBL in a qualitative sense. However, due to the simplicity of our set-up, quantitatively DNS results provide an order-of-magnitude estimate only. Beyond the critical point, that is in the VSBL, no direct relation to atmospheric flows should be made, other than that a qualitative regime transition occurs. In other words, MO similarity predicts that a regime transition occurs, not what happens beyond this transition. Detailed analysis of high-stability Couette flows is more suited to configurations using a fixed ambient stability, such that intermittent behaviour is observed as a quasi-steady state (e.g. Deusebio et al. 2015). For the comparison to the real VSBL a suitable approach would be to extend Ekman flows (such as in Ansorge and Mellado 2014) with a surface energy model.

A notable simplification is the reduction of the Reynolds number to several orders of magnitude below the atmospheric case. Whereas $Re_L$ (Flores and Riley 2011) explicitly depends on fluid viscosity (or *Re* in dimensionless terms), $SC_C$ does not. This can be explained by the fact that, at high *Re*, diffusive transport is negligible in the flow centre. Therefore, turbulent heat transport must adjust to the boundary conditions to attain a fully turbulent steady state. Once turbulent heat transport is insufficient, the positive feedback mechanism propels the system to strongly stratified conditions. Because the current *Re* is relatively low, some quantitative effects may be expected, though this is not systematically investigated here. Nonetheless, an indication may be obtained from the relative contribution of the diffusive heat transport to the total heat transport in the centre of the flow. The magnitude of this contribution in Fig. 4 (a few per cent) suggests that low-*Re* effects on $SC_C$ are small, though not negligible, but they do not alter our main conclusions.

## Summary and Outlook

Using an idealized system we showed that a collapse of turbulence occurs when the wind speed is less than a minimum set by surface cooling or, alternatively, if the extracted heat flux is larger than the maximum set by the wind speed. This result confirms previous analytical (Wiel et al. 2012b) and idealized single-column model results (Wiel et al. 2007). Furthermore, the results are consistent with those of other numerical investigations (Nieuwstadt 2005; Flores and Riley 2011; Donda et al. 2015b) and observational studies (Sun et al. 2012; Monahan et al. 2015; Hooijdonk et al. 2015). Additionally, generic (i.e. slowing down) and specific (i.e. system response) early warning signals were found to be present in the turbulent flow field. As shown, a quantitative estimate of the critical point could be inferred from specific signals. Our results can be seen as a proof of principle that early warning signals for regime transition are present in stably stratified turbulent flows. Extending this metric to field observations will be challenging, though as these show more significant scatter. Finally, we observed slowing down when surface cooling was increased. On the other hand, results with respect to variance leave room for further research on how these potential tools manifest themselves in turbulent flows.

## Figures and Tables

**Fig. 13:**
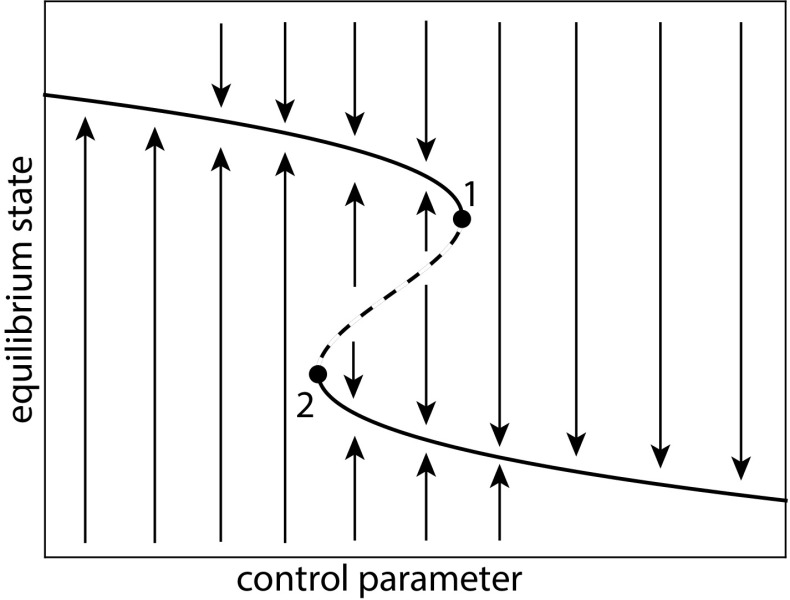
Sketch of the steady state of a dynamical system with hysteresis. *Arrows* indicate dynamical behaviour. Stable equilibria are indicated with a *solid line*, unstable equilibria are indicated with a *dashed line*. *Solid dots* denote transition point 1 and 2. Adapted from Scheffer et al. (2001)

## Appendix: Critical slowing down

Here, we base ourselves on the introductions to bifurcation theory by Seydel (2010) and Scheffer (2009) to introduce some key concepts. The equilibrium state of a dynamical system is generally a function of one or more external conditions. For example, the total incoming solar radiation controls the global temperature (on a glacial time scale). Small changes in solar radiation typically lead to a proportional change in the Earth’s temperature (upper branch in Fig. 13). On the other hand, if the temperature changes owing to a unique event, the original state is recovered after some time; the equilibrium is dynamically stable. If, however, the solar radiation decreases beyond a critical point (cf. point 1 in Fig. 13), the Earth’s temperature may drastically change to an alternative state. This is the result of a positive feedback between temperature (i.e. ice coverage) and albedo. In this example, the system also contains hysteresis: if solar radiation increases again, the system will not transition back to the warm state immediately. Only if solar radiation increases beyond a second critical point does a return transition occur (cf. point 2 in Fig. 13). As a result, for some range of external conditions, two alternative, dynamically stable states exist: a moderate climate and the (completely ice-covered) ‘snowball’ Earth. These stable states are separated by an unstable equilibrium. Above the dashed line, the system will move towards the upper branch, while below the dashed line, the system will move towards the lower branch. Alternatively, perturbations with respect to the unstable (dashed) branch will grow in time, while with respect to the stable branches (solid) perturbations diminish over time. From Fig. 13, we observe that at both critical points, stability changes from stable (solid) to unstable (dashed). Thus, at the critical point, perturbations neither grow or diminish. As such, when the critical point is approached, the rate at which perturbations diminish decreases, that is it takes longer to recover from perturbations near the critical point. The growing recovery time scale is named critical slowing down.
